# Editorial: Ion channels & homeostasis of ions in cancer cell fate

**DOI:** 10.3389/fonc.2022.1090583

**Published:** 2022-12-22

**Authors:** Wei Cheng, Linlin Ma, Zui Pan

**Affiliations:** ^1^ Institute of Cancer Stem Cell, Dalian Medical University, Dalian, Liaoning, China; ^2^ Griffith Institute for Drug Discovery, Griffith University, Nathan, Brisbane, QLD, Australia; ^3^ School of Environment and Science, Griffith University Nathan, Brisbane, QLD, Australia; ^4^ College of Nursing and Health Innovation, The University of Texas at Arlington, Arlington, TX, United States

**Keywords:** ion channels, ion homeostasis, cancer, cell fate, pathogenesis

In the last several decades, ion channels in a plethora of fundamental cell functions and their roles in hereditary and diseases have been extensively explored. Channelopathies are usually defined as diseases caused by inherited mutations that alter ion channel biophysical properties. In recent years, it has been expanded to disorders caused by dysregulated ion channel expression, membrane trafficking, and/or posttranslational modifications. Dysregulation of ion channels in distribution or gating, and consequent distortion of ion homeostasis have been found implicated in a variety of cellular process, including proliferation, cell migration, cell survival, and programmed cell death which in turn, affect cancer cell fate (1–4). In that sense, cancers caused by altered ion channel expression and/or activity may be classified into the channelopathy family (5).

The present Research Topic entitled “*ion channels & homeostasis of ions in cancer cell fate*” aimed to collect the latest advancements in ion channels and ion homeostasis participating in the regulation of cancer cell fate. The total collection brings together unique contributions from original research to review articles covering the dysregulation of K^+^, Ca^2+^, Mg^2+^, iron and water by a variety of channels in multiple cancers.

Inwardly rectifying potassium channel GIRK1 (KCNJ3) has been reported to play a role in the pathogenesis and progression of breast cancer (6). Pelzmann et al. carefully selected 15 missense mutations within the pore region of GIRK1 (out of 304 documented somatic mutations of KCNJ3 gene in primary tumors) and investigated how these primary cancer-associated mutations change the expression and functions of the channel. Interestingly, they found that gain-of-function mutations may play a pathological role in cancers. Also on the topic of breast cancer, Charlestin et al. reviewed the aquaporins which selectively transport water and other small molecules and ions, acting as new players in breast cancer progression and treatment response.

Gastrointestinal cancer is even more challenging to be identified than breast cancer, accounting for about 20% of all diagnosed cancers and nearly 30% of cancer deaths. Increased risk of gastric and colorectal cancer has been reported to be associated with the administration of proton pump inhibitors (PPI), but the underlying mechanisms are elusive (7). In this Research Topic collection, Kampuang and Thongon correlated this observation with PPI-induced hypomagnesemia (PPIH) (8, 9), and investigated the potential roles of non-selective cation channels of TRPM6 & TRPM7 in the related pathological mechanisms. Using a rat model of PPIH, it was demonstrated for the first time that TRPM6/7 heterodimers, which allow continuous Mg^2+^ absorption, are expressed in the duodenal and jejunal epithelial plasma membranes, while their level is reduced in the small intestine of PPIH rats. Altered phosphorylation patterns of TRPM6 and TRPM7 channels in PPIH, which affect channel permeability and stability, may also contribute to this pathological condition. These intriguing findings add to the already diverse physiological and pathological functions of the TRPM6 and TRPM7 channels (10).

Ferroptosis is a novel form of cell death characterized by the accumulation of iron-dependent lipid reactive oxygen species (ROS) and disruption of plasma membrane unsaturated fatty acids (11, 12). Recently, ferroptosis has been implicated in the pathogenesis of cancer. Particularly, many genes have been found to play vital roles in breast cancer by regulating ferroptosis. Wu et al. developed a prognostic prediction model with 15-ferroptosis-related gene features based on a calculation of the risk score of triple-negative breast cancer (TNBC) patients and matched clinical data from The Cancer Genome Atlas (TCGA) database.This prediction model may inspire a new avenue for the treatment of cancer by targeting ferroptosis. On the same topic of ferroptosis and cancer, Ke et al. reviewed the regulatory roles and crosstalk of Fe^2+^, Ca^2+^, Zn^2+^, Cu^2+^, Se^2+^ cations and Cl^-^ anions in ferroptosis, and proposed the potential application value of iron and other ions in the treatment of ferroptosis-associated cancers.

Physiological and pathological stimuli could dynamically change the abundance of ion channels in the plasma and intracellular membranes. Capera et al. investigated the mitochondrial translocation pathway of the voltage-gated potassium channel Kv1.3, and proposed a mechanism regulating the distribution of Kv1.3 in mitochondria, which is involved in mediating cell survival and apoptosis.

Ion channels play important roles in almost all aspects of cancer biology ranging from tumor microenvironment and cancer metabolism to cancer stem cells, even cancer chemo-resistance (Zhao et al.), which evenually affect cancer cell fates (**Figure 1**). Therefore, the study of ion channels in cancer has become a fascinating scientific field, attracting more and more exciting studies. The present Research Topic collection only depicts one facet of ion channels in cancer cell fate. Further research exploring the complexity of ion channels in cancers will help us solve the mystery of highly complex cancer pathogenesis. We hope that this special issue will provide new insights into the current understanding of ion channels and different ions involved in cancers.

**Figure 1:**
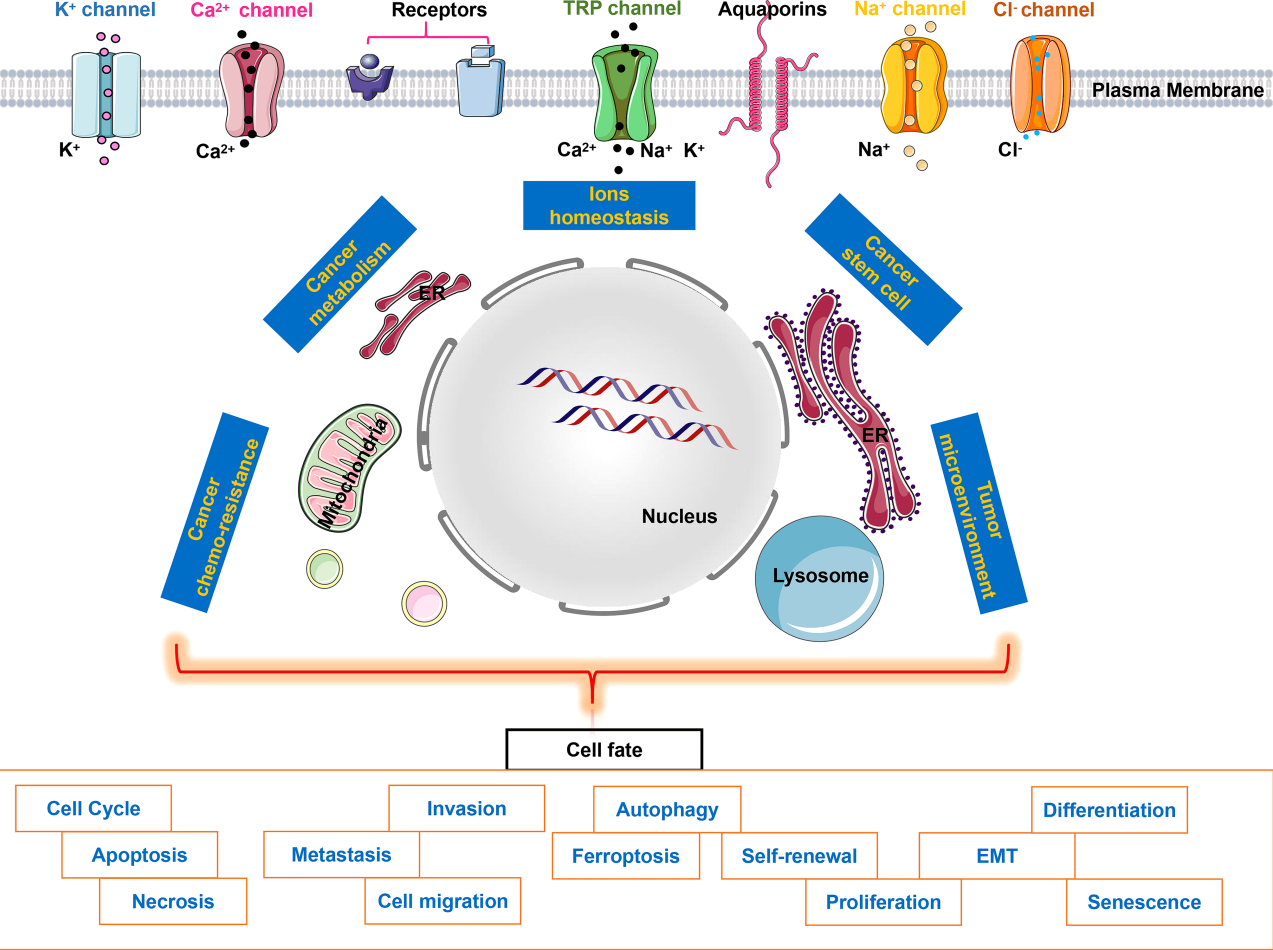
Ion channels & ion homeostasis in cancer cell fate. Intracellular ion fluctuations triggered by specific stimuli of ion channels activate downstream signaling cascades, which affect ions homeostasis, cancer metabolism, cancer stem cell, cancer chemo-resistance or tumor microenvironment, therefore determining cancer cell fate eventually.

## Author contributions

All authors listed have made a substantial, direct, and intellectual contribution to the work and approved it for publication.
